# Severe clinical immunodeficiency in a patient with human immunodeficiency virus infection and relatively high CD4 counts: a case report

**DOI:** 10.1186/s13256-019-1982-2

**Published:** 2019-03-14

**Authors:** Mickael Essouma, Larry N. Tangie, Mazou N. Temgoua, Ulrich Gabin Kenfack, Antonin N. Ndam, Celestin Danwang

**Affiliations:** 1 Department of Internal Medicine and Specialties, Faculty of Medicine and Biomedical Sciences, University of Yaounde I, Yaounde, Cameroon; 2 Hepatogastroenterology Unit, Yaounde General Hospital, Yaounde, Cameroon; 3 Department of Surgery and Specialties, Faculty of Medicine and Biomedical Sciences, University of Yaounde I, Yaounde, Cameroon

**Keywords:** HIV infection, CD4 count, Immunodeficiency, Gastric Kaposi’s sarcoma, Neuromeningeal cryptococcosis

## Abstract

**Background:**

The coexistence of neuromeningeal cryptococcosis and Kaposi’s sarcoma is not surprising in a patient with human immunodeficiency virus infection and a low CD4 count, although it is rarely described. However, we describe such an association in a patient with human immunodeficiency virus infection and a relatively high CD4 count.

**Case presentation:**

A 41-year old Cameroonian woman presented to our hospital with subacute occipital headaches associated with photophobia, blurred vision, phonophobia, projectile vomiting, and tonic seizures. In her past history, there was an human immunodeficiency virus infection known for 12 years, for which she had been taking (with good compliance) tenofovir-lamivudine-efavirenz-based antiretroviral therapy for the same period of time. One month before the consultation, gastric Kaposi’s sarcoma had been diagnosed, justifying the treatment with doxorubicin she had received. A clinical examination was unremarkable. A computed tomography scan of her brain was normal, and cerebrospinal fluid analysis revealed *Cryptococcus neoformans*. Her CD4 count was 353/mm^3^. Orally administered antifungal treatment with fluconazole (1200 mg/day) and flucytosine (1500 mg × 4/day) was started immediately, but she died on the sixth day of this treatment.

**Conclusion:**

This clinical case shows that the coexistence of neuromeningeal cryptococcosis and gastric Kaposi’s sarcoma is possible in all patients with human immunodeficiency virus infection, regardless of CD4 count.

## Background

Since the introduction of highly active antiretroviral therapy (HAART) worldwide in 1996, there has been a decline in mortality from human immunodeficiency virus (HIV) infection. This is mainly due to a decrease in opportunistic diseases [1]. Neuromeningeal cryptococcosis is an opportunistic infection caused by a ubiquitous environmental encapsulated yeast (*Cryptococcus* species), usually seen in patients with HIV infection and advanced immunodeficiency (CD4 count < 100 cells/μl) [2]. Kaposi’s sarcoma is another opportunistic disease associated with severe immunodeficiency, mostly occurring in individuals with HIV infection and a CD4 count < 100 μl, even though it can be seen at any stage of the disease [3].

Simultaneous diagnosis of neuromeningeal cryptococcosis and Kaposi’s sarcoma is not surprising in a patient with HIV infection and low CD4 counts, although this is rarely reported. [4]. In this article, we describe the coexistence of neuromeningeal cryptococcosis and gastric Kaposi’s sarcoma in a patient with HIV infection and a relatively high CD4 count, who had a fatal outcome.

## Case presentation

**Fig. 1:**
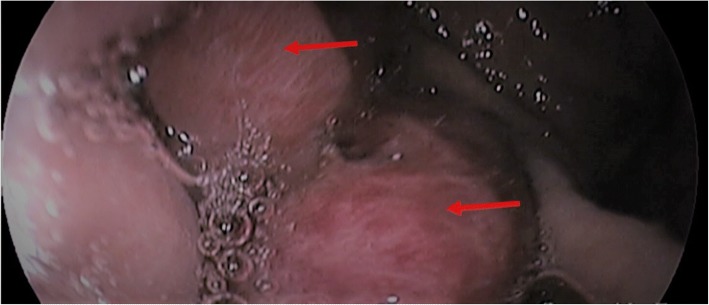
Upper endoscopy showing infiltration of the gastric body by nodules of Kaposi’s sarcoma (*arrows*)

A 41-year-old Cameroonian woman was seen in a hospital for progressively worsening occipital headaches of 4 weeks’ duration, associated with phonophobia, photophobia, blurred vision, projectile vomiting, and tonic seizures. In her past history, there was an HIV infection known for 12 years and for which she had been taking (with good compliance) tenofovir-lamivudine-efavirenz-based antiretroviral therapy for the same period. No opportunistic diseases had been noted in the last 11 years. One month prior to the consultation, an upper endoscopy (Fig. 1) performed to investigate persistent gastric pain led to the diagnosis of gastric Kaposi’s sarcoma. She had already received a systemic chemotherapy cycle with doxorubicin. Upon admission, her clinical status revealed an ill-looking and alert (Glasgow Scale E4V5M6) patient. Her parameters were: temperature 37.8 °C, blood pressure 176/120 mmHg, pulse rate 153 pulsations/minute, respiratory rate 25 cycles/minute, and weight 59 kg. A computed tomography (CT) scan of her brain with injection of contrast products was normal. Cerebrospinal fluid (CSF) analysis revealed: protein 1.2 g/l, glucose 0.54 g/l (concomitant glycemia 1.25 g/l), leukocyte 1/mm^3^, positive Indian ink stain and culture for *Cryptococcus neoformans*, and negative GeneXpert for acid-fast bacilli. Analysis of blood tests revealed: hemoglobin 9.6 g/dl, white blood cell count 2120/mm^3^, lymphocytes 848/mm^3^, platelets 604,000/mm^3^, CD4 count 353/mm^3^ (350–1600), C-reactive protein 48 mg/l, blood urea nitrogen 0.15 g/l, and creatinine 7 mg/l. Considering the diagnosis of neuromeningeal cryptococcosis, orally administered antifungal treatment with fluconazole (1200 mg/day) and flucytosine (1500 mg × 4/day) was started immediately. During hospitalization, her blood pressure normalized without treatment, but she died on the sixth day of antifungal therapy after a rapid deterioration of the state of conciousness.

## Discussion

We describe the coexistence of neuromeningeal cryptococcosis and gastric Kaposi’s sarcoma in a patient with HIV infection and a CD4 count of 353. These are two conditions that classify patients as having acquired immunodeficiency status of HIV infection, and therefore are often found in patients with low CD4 count. The major limitations of this work are the absence of concomitant HIV viral load which is the direct measure of HIV activity, and the lack of evidence of human herpesvirus 8 (HHV-8) infection.

Neuromeningeal cryptococcosis is the most common presentation of cryptococcal disease that classically develops in patients with HIV infection and CD4 counts < 100/μl [2]. Other risk factors include organ transplantation, long-term corticosteroid use, chronic kidney and liver diseases, autoimmune/chronic inflammatory diseases, and idiopathic CD4+ lymphopenia. Since *Cryptococcus* species are optional intracellular germs, the increased risk of neuromeningeal cryptococcosis in these patients is mainly due to immunodeficiency affecting cellular immunity [5]. In fact, extrapulmonary infection with *Cryptococcus* species would be by an active mechanism of phagosomal extrusion, allowing them to spread from macrophage to macrophage. This hypothesis of spread of *Cryptococcus* species through macrophages is called the “Trojan horse” model, and the “Trojan horse” crossing of the blood–brain barrier is thought to be the main mechanism underlying neuromeningeal cryptococcosis. Beyond this general mechanism, other intrinsic factors and cryptococcal virulence may have been implicated in this patient. These intrinsic additive factors are essentially host molecules (protein CD44, protein kinase C, ganglioside GM1, and dual specificity tyrosine phosphorylation-regulated kinase 3 which is abbreviated to DYRK3) interacting with fungal ligands (for example, hyaluronic acid), while the virulence factors of *Cryptococcus* facilitating the crossing of the blood–brain barrier are cellular morphology (small cells) and the biochemical structure of the capsule (presence of polysaccharides, mannitol, melanin, phospholipase, prostaglandins, and urease) [6].

As observed in this patient, the classic signs of meningitis are often absent during neuromeningeal cryptococcosis in a patient with HIV infection. Cryptococcal meningitis is typically a lymphocytic meningitis. However, this patient presented a poor CSF inflammatory response (1 leukocyte/mm^3^) limiting conclusions in this regard. Of note, poor CSF inflammatory response is more frequent in patients with HIV infection and CD4 count < 50/μl [4]. Three molecules are recommended for the treatment of neuromeningeal cryptococcosis: amphotericin B, flucytosine, and fluconazole. This treatment takes place in three phases: induction (2 weeks), consolidation (8 weeks), and maintenance. During the induction phase, it is recommended to combine amphotericin B deoxycholate (1 mg/kg per day) with flucytosine (100 mg/kg per day) for 1 week, followed by fluconazole (1200 mg/day for adults and 12 mg/kg per day for children and adolescents up to a daily dose of 800 mg) in monotherapy for 1 week [7]. In developing countries (including those in sub-Saharan Africa) where amphotericin B is not available, the best therapeutic alternative during the induction phase is the combination of fluconazole (1200 mg/day in adults and 12 mg/kg per day in children and adolescents up to a daily dose of 800 mg) with flucytosine (100 mg/kg per day) for 2 weeks [7, 8]. The choice of alternative treatment in this patient was based on this scientific evidence. Fluconazole is the only molecule recommended for the consolidation and maintenance phases of treatment.

Cryptococcal meningitis is associated with a bad prognosis, accounting for an estimated 15% of all deaths related to HIV infection globally, three quarters of which occur in sub-Saharan Africa [7]. Risk factors for mortality in patients with cryptococcal meningitis are: high CSF fungal load, CD4 count < 100 cells/μl, poor CSF inflammatory response (< 20 leukocytes/mm^3^), and delayed diagnosis and treatment [4]. In untreated patients, further contributors to mortality are complications of raised intracranial pressure as well as immune reconstitution inflammatory syndrome associated with cryptococcal meningitis and HAART [7]. Although the patient did not have a low CD4 count and the antifungal treatment was administered promptly, the combination of other poor prognostic factors could, at least in part, explain the patient’s death.

Kaposi’s sarcoma is a malignant tumor of the vascular endothelium caused by the HHV-8 virus of the *Herpesviridae* family. This tumor can affect the skin and viscera; gastrointestinal involvement being the most frequent visceral involvement. Four clinical forms of Kaposi’s sarcoma are described: (i) classical form (rare visceral involvement), (ii) endemic form (often found in Africa, mainly characterized by skin involvement), (iii) iatrogenic form (related to drug immunosuppression) and (iv) the epidemic form (related to immunosuppression by HIV, characterized by cutaneous involvement that may progress to visceral involvement at the late stage) [3]. Taking into account this classification, the most likely hypothesis was that of an epidemic Kaposi’s sarcoma. What is surprising, however, is that our patient had a relatively high CD4 count and she had no cutaneous signs. Most patients have cutaneous signs and a CD4 count of less than 100 at the time of diagnosis of gastric Kaposi’s sarcoma [3]. In such a patient with a relatively high CD4 count, the mechanisms that can explain Kaposi’s sarcoma are, on the one hand, the interaction between HIV and HHV-8, and on the other hand, the interaction between HIV proteins (e.g. Tat) and host cell molecules (basic fibroblast growth factor, arginine-glycine-aspartic acid binding integrins). These hypotheses assume evidence of HHV-8 infection and high HIV viral load, so increased viral activity [9]. The combination therapy regimen of HAART with doxorubicin that we used in this patient provides the best benefit in terms of survival for patients with gastric Kaposi’s sarcoma but therapeutic failure is observed in the third of patients [3]. 

Co-occurrence of Kaposi’s sarcoma and cryptococcal meningitis in the setting of HIV infection is rare, as illustrated by an unusual case of both diseases in a South African cohort of 127 individuals with HIV infection and low CD4 count [4]. The coexistence of both pathologies in this patient strongly suggested an acquired immunodeficiency syndrome, despite a relatively high CD4 count.

## Conclusions

From this clinical case, there is evidence that both cryptococcal meningitis and gastric Kaposi’s sarcoma can coexist at any stage of HIV infection, and irrespective of the CD4 count. A clinical suspicion for these diseases should still be maintained and a low CD4 count should not stop testing for the same.

## Abbreviations

CSF: Cerebrospinal fluid
HAART: Highly active antiretroviral therapy
HHV-8: Human herpesvirus 8
HIV: Human immunodeficiency virus
